# Synthesis of biodiesel from *Annona muricata – Calophyllum inophyllum* oil blends using calcined waste wood ash as a heterogeneous base catalyst

**DOI:** 10.1016/j.mex.2020.101188

**Published:** 2020-12-23

**Authors:** T.F. Adepoju

**Affiliations:** Akwa Ibom State University, Ikot Akpaden, Mkpat Enin L.G.A, Nigeria

**Keywords:** Biodiesel, Heterogeneous catalyst, Blending, Optimization, Physicochemical properties

## Abstract

Naturally, biodiesel synthesized from highly viscous and high-density vegetable oil is usually unsuitable as fuel in the internal combustion engine. However, mixing/blending of two or more oils as a feedstock for biodiesel production could produce a low viscous fuel suitable for the engine. This study produced a novel heterogeneous base catalyst from waste wood ash (WWA) and applied it to synthesis of biodiesel from *Annona muricata* and *Calophyllum inophyllum* oilseed blend. The production route was via a two-step process due to the high free fatty acid of the blended oil. Process optimization of the transesterification step was carried out via response surface methodology (RSM). The strength of the developed catalyst was tested through catalyst regeneration and recyclability. The quality of the biodiesel was compared with biodiesel recommended standard.

• Waste wood ash contained a high percentage of calcium carbonate
• Blended oil produced oil of low viscosity
• Two-step production route was used for biodiesel synthesis
• Process optimization via hybrid design produced optimum biodiesel yield.

## Graphical abstract

**Figure:**
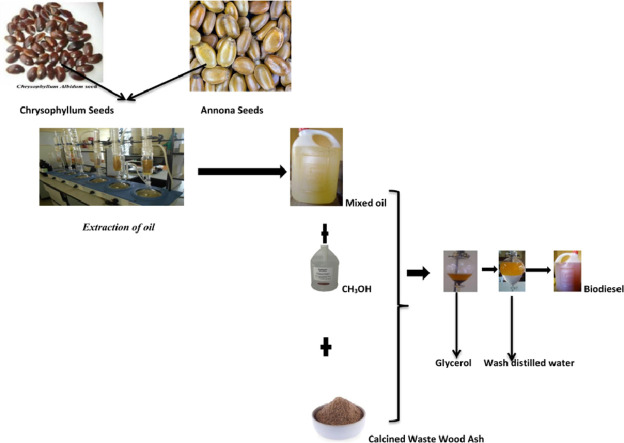

**Specifications Table**

**Table:** 

Subject Area	Energy
More specific subject area	Biodiesel
Method name	API gravity blend ratio, Catalyst characterization, transesterification
Name and reference of the original method	Synthesis of biodiesel from the binary blend of *Annona muricata –Calophyllum inophyllum* oil using fly ash
Resource availability	n/a

## Background

Renewable, but sufficiently considered as ecofriendly, nontoxic, economically competitive, and environmentally beneficial are terms used to describe the liquid biodiesel (biofuel) derived from biomass sources that can be synthesized via biological routes in the presence of suitable catalyst [1].

The base catalyst used for biodiesel synthesis is classified mainly as a homogeneous catalyst (hydroxide of Na/K) and heterogeneous catalyst (majorly from solid wastes). The work reported by Ozkan et al. [2] developed calcium oxide (CaO) from waste materials as a heterogeneous catalyst for the synthesis of biodiesel, while [3] employed the use of bio-based catalyst from cocoa pod husk as a heterogeneous catalyst for biodiesel production. Ogunkunle et al. [4] reported the production of biodiesel using both catalysts. The work of [5] produces biodiesel with the help of a heterogeneous catalyst, while [6] described the use of a heterogeneous catalyst in the optimization of the production of rubber seed/palm oil biodiesel, IDI diesel engine performance, and emissions. Milano et al. [7] further used heterogeneous catalysts for the conversion of waste cooking oil-Calophyllum inophyllum to biodiesel, but the work reported by Nath et al. [8] utilized the green heterogeneous base catalysts as an effective bio-based for the conversion of oil to biodiesel. Papasanee et al. [9] reported a novel process for biodiesel production from slum sludge whereas [10] studies the use of catalyst derived from cotton stalk for production of biodiesel from Madhuca indica oil. In another study, Choksi et al. [11] reported the development of catalyst derived from palm-fruit-bunch for biodiesel. However, the conversion of a low-value industrial waste into biodiesel via a catalyst derived from brewery waste was reported by Saravanan et al. [12]. The advantages of using heterogeneous catalysts include ease of recoverability and reusability, readily available, nontoxic, eco-friendly, and low cost [13,14].

Among the low-cost base catalysts is the waste wood ash (WWA), this is a fine powder that is a byproduct of combustion of wood such as burning wood in a home fireplace or an industrial power plant. Researchers have revealed that the major components of the waste wood ash are calcium carbonate (CaCO_3_) [15], some find no carbonate but calcium oxide (CaO) [16], some show the presence of about 12% iron oxide due to soil contamination while some show none [17]. However, because of its availability, environmental pollution effects, non-weather resistance, unsuitability for polishing, and brittle structure, it is considered as base-feedstock for biodiesel synthesis from seed oil.

Biomass sources of feedstock for biodiesel production could be derived from vegetable oil or animal fat [9]. The oil could be edible or non-edible oil, depending on the acid value of the oil. Oil can be classified as edible if the acid value of the oil is less than 3.00 mg KOH/g oil (%FFA < 1.5), and can successively undergo transesterification reaction with a base, while the non-edible oil acid value must be greater than 3 mg KOH/g oil (%FFA ≥ 1.5), which required acid esterification (H_2_SO_4_/HCl) before transesterification with base.

Meanwhile, many fruits bearing seeds are produced all over the nation from under-utilized to the most utilized one. These seeds majorly constitute a nuisance to the environment, which results in environmental pollution due to the problem of disposal. Among the fruits producing seeds are *Annona muricata* (Soursop) and the C*alophyllum inophyllum (*Berry). Studies revealed that soursop seed is rich in oil ranging from 22.57% to 34.61%, the oil nature proved edible and contained high unsaturated compounds [18,19]. Berry on the other hand was reported to be rich in oil up to 75% and contained both linoleic (36.0) and oleic (37.6%) fatty acid [14,20].

Response Surface Methodology (RSM) is an optimization software package for variable optimization of complex processes that helps to understand the interaction of the variables, produce several experimental runs, determine the level of significance, and predict the optimum conversion of the output. This software has been reported to apply to biodiesel synthesis from various oil feedstocks [13,^14^,21]. The software is capable of handling design related factors such as Central Composite Design (CCD), Central Composite Rotatable Design (CCRD), Box Behnken Design (BBD), Pentagon, Hexagon, d-Optimal, Distance-based, Modified Distance, 3 -Level Factorial, Hybrid, One-Factor, User Design, and Historical Data. When dealing with four process variable factor with three/five levels, only CCD and Hybrid design can be exploited. Since CCD can give a higher number of experimental runs (30) with repetition runs, presence of lack of fit, and give room for outliers, Hybrid design (HD) is preferable to overcome the CCD drawback. HD can easily produce 16 runs with no lack of fit, no repetition, and does not give room for outliers.

Hence, this paper exploits the use of blended oil of *Annona muricata* and *Calophyllum inophyllum* for the synthesis of biodiesel in the presence of waste wood ash (WWA) as a base-catalyst. Blend oil physicochemical properties were determined through AOAC, 1997 [22] standard methods, catalyst characterization of WWA were determined using scanning electron microscopy (SEM), energy dispersive spectroscope (EDS), X-ray diffraction analysis (XRD), Fourier transforms infrared spectroscopy (FTIR), BET isothermal adsorption, and Hammett indicators. The quality of the biodiesel was ascertained by comparing it with biodiesel standard (ASTM D6551 [23] and EN 14,214 [24]).

## Experimental

### Oil extraction and its physicochemical properties

Oil was extracted from the cleaned milled seed powder of *Annona muricata* and *Calophyllum inophyllum* using a soxhlet extractor with n-hexane as solvent. The reaction temperature was set at the range of the boiling point of n-hexane, and the reaction time was maintained at 70 min for complete oil extraction from the two powders. The excess n-hexane in the oil was recycled and reused, while the residual cake was used as an animal feed supplement. The physicochemical properties of the two oils were determined to ascertain the suitability of the oil for biodiesel synthesis.

### Oils blending

The oil’ blending was carried out based on the physicochemical properties of the oil depicted in Table 1. Since the most significant parameters needed to achieve volatile mixed oil are the viscosity, the specific gravity (density), and the API gravity ratio of the oil. In this case, the viscosities of the oils were determined using a viscosimeter and the specific gravity of the oil by using a specific gravity bottle. The API gravity of the oil was estimated using the equation already adopted by Adepoju [25] as listed in Eq. (1).

(1) $$API\;gravity=\frac{131.5\left(1.076-SG\right)}{SG}$$

Where: SG is the specific gravity of the oil; API gravity is the American Petroleum Institute gravity.

**Table 1:** Properties of oils and blended oil.

Properties	AMO	CIO	Mixed/Blended oil	Total API gravity
Moisture content (%)	0.011	0.011	0.001^a^	
Viscosity @ 40 °C/ (mm^2^/s)	2.32	5.40	3.35^a^	
Acid value (mg KOH/g oil)	1.56	6.84	4.10^a^	
% Free Fatty Acid (FFA)	0.78	2.92	2.05^a^	
Peroxide value (meq O_2_/kg oil)	1.34	1.40	ND	
Saponification value (mg KOH/g oil)	224.63	201.00	ND	
Iodine value (g I_2_/100 g oil)	114.32	68.56	ND	
Specific gravity	0.82	0.91	ND	
API gravity	41.05	24.00	ND	
API gravity ratio (%)	63	37		
Simplest Ratio	1.7	1		
**Blended ratio**	**AMO_63_: CIO_37_**			

where, am = Value after mixed/blended, NYD = Not Determined.

Hence, the mixed/blended ratio of the oil was computed by adding up the API gravity of oils, resulting in the total API gravity as stated in Eq. (2) (supplementary file). The blend ratio (BR) of the oil was estimated by dividing the API gravity of individual oil with the total API gravity in Eq. (2), as estimated in Eq. (3). The blended oil obtained was preheated on a magnetic shaker at 50 °C to achieve a homogeneous phase with low viscosity and specific gravity required for biodiesel production.

(2) $$API\;gravity_{Total}=\;SG_{AMO}+SG_{CIO}$$

(3) $$BR=\left(\frac{SG_{AMO}\;}{API\;gravity_{Total}}X100:\;\frac{SG_{CIO}\;}{API\;gravity_{Total}}X100\;\right)$$

Where $API\;gravity_{Total}$ is the total API gravity, $SG_{AMO}$ is the specific gravity of *Annona muricata,* $SG_{CIO}$ specific gravity of *Calophyllum inophyllum.*

The% free fatty acid (%FFA) of the oils and the blend was determined using a standard titration method:

0. 1 M of 95% ethanolic potassium hydroxide (KOH) was titrated against the mixture containing 2.0 g of oil in a hot mixture of ethanol and petroleum ether (1:1), with two drops of phenolphthalein as an indicator (Table 1).

### Catalyst preparation and characterization

The obtained WWA from the local bakery was soaked in distilled water for 30 mins to remove unwanted particles floating on the surface of the sediment. The unwanted particles were removed by decantation, while the pure wetted waste wood ash (PWWWA) was obtained through filtration. The filtrate was discarded, and the residual PWWWA was oven-dried at 110 °C in an oven until a constant weight is achieved. The dried WWA was calcined in a programmable muffle furnace at 1100 °C for 3 h, the calcined waste wood ash (CWWA) was allowed to cool to room temperature for 48 h, before characterization.

**Fig. 1:**
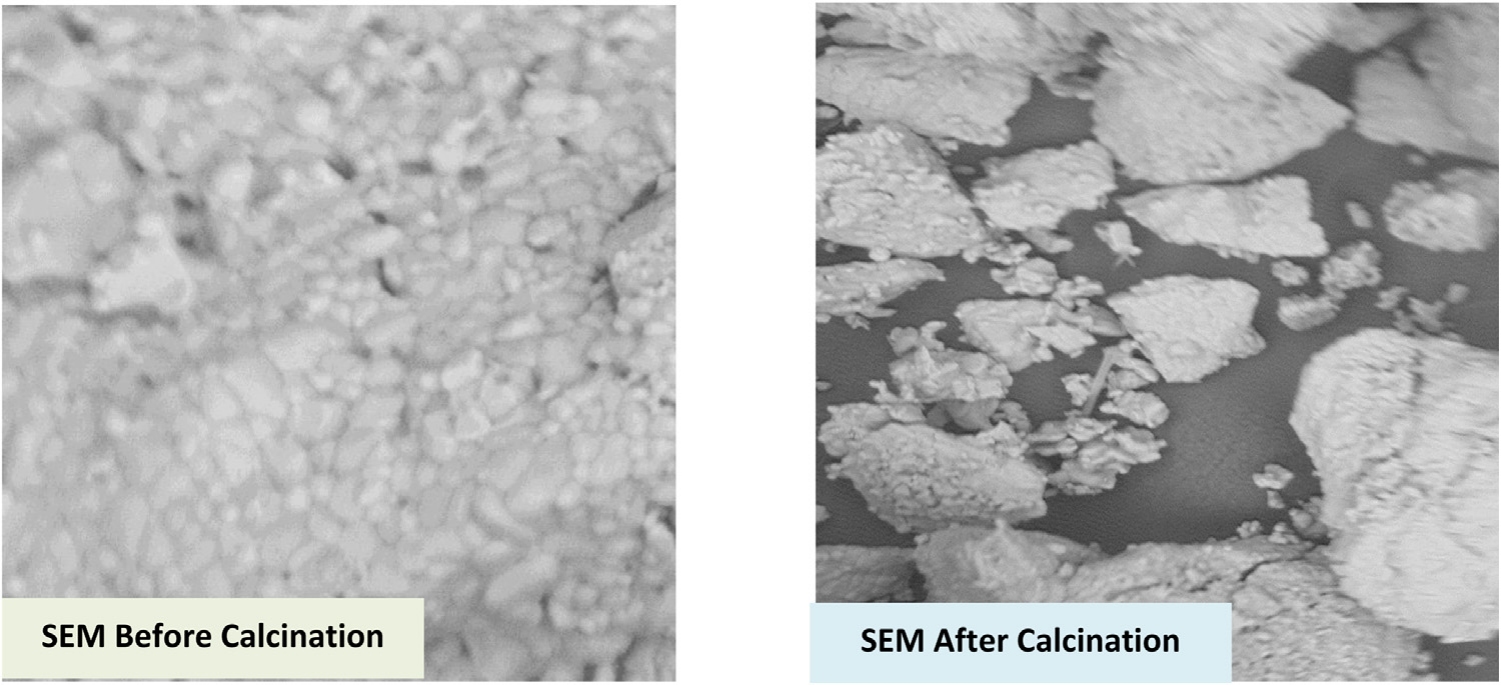
SEM analysis of the WWA.

**Fig. 2:**
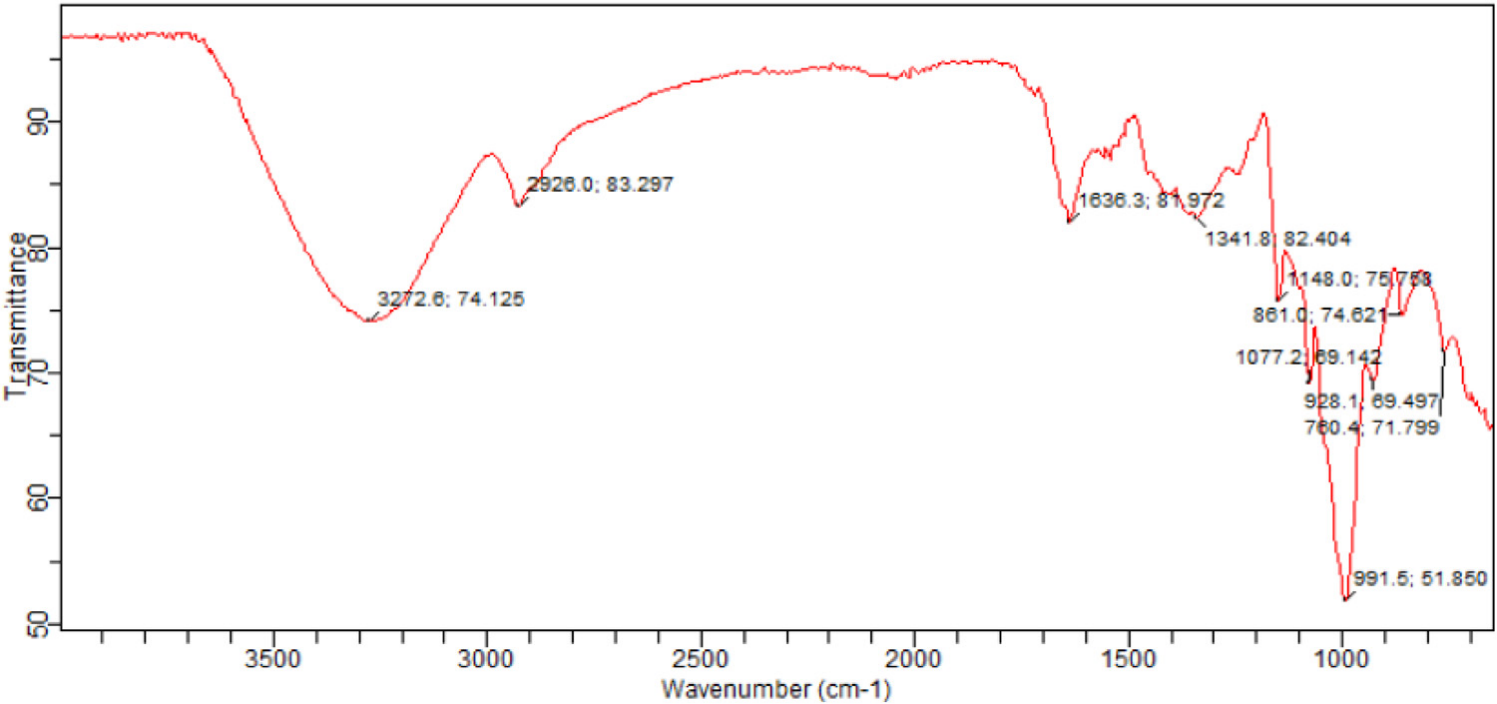
FTIR spectral of CWWA.

Characterization of the CWWA and WWA was carried out using scanning electron microscopy (SEM) to determine the wide range of surface topography and composition illustrated in Fig. 1. Fourier transforms infrared spectroscopy (FTIR) to determine the chemical bonds and the angle of resolution within the wavelength band displayed in Fig. 2. The spectra give rise to the shape of the CWWA and WWA as an individual molecular pattern that can be used to screen and scan the powders for different transmittances as explained in Table 2. X-ray diffraction analysis (XRD) was used to quantitative and qualitatively estimated the elemental compositions in the sample. BET isothermal adsorption to determine the surface area, the pore volume, and the total basic density of the powder, while Hammett indicator was used to determine the basic strength of the CWWA displayed in Table 3.

**Table 2:** FTIR sample spectrum analysis of CWWA.

SN	Wavelength (cm^−1^)	Transmittance (%)	Bonding Functional groups
1	760.4 to 1077.2	71.799 to 69.142	C-Cl, CO_3_^2−^, N—H waging and twisting, O = C = O bending vibration
2	1148.0 to 1636.3	69.142 to 81.972	C—C, C = C, C = N, C = O, CHO, C≡C, C≡N, O = C = O of low energy, O—H, and O—Ca-O bending vibration
3	2928.0 to 3272.0	83.297 to 74.128	O-H bending structure, O = O, and N≡O

**Table 3:** BET-adsorption, XRD analysis, and Hammett indicator value of calcined CWWA.

Catalyst	N_2_-AA(m^2^g^−1^)	TPV(cm^3^g^−1^)	%CaO	BS (μmole.g^−1^)400<BS<500 >500		TBS	BSD (μmole/m^2^)
*CWWA*	1.10	0.0045	62.83	47	162	209	190.00

N_2_-AA= nitrogen adsorption analysis, TPV = Total pore volume, BS = Basic site, TBS = Total basic site, BSD = Basic site density.

## Biodiesel production

### Esterification: acid treatment

The acid value of the blended oil was determined to be 4.10 mg KOH/ g oil (%FFA = 2.05), acid value reduction was carried out through the esterification process using a 250 ml three-necked reactor in a temperature control hot plate with a magnetic stirrer. The reaction temperatures were varied between 50 and 90 °C at a reaction time of 40–80 min with H_2_SO_4_ concentration of 1.0–3.0% (v/v). A total of five experiments (Table 4) were performed to establish a perfect process condition for the lowest acid value of 2.02 mg KOH/g oil (%FFA = 1.01). A known amount of acid concentration was mixed with methanol in the ratio of blended oil/methanol ratio, the reaction was allowed till completion at a specific temperature and time. At the end of the reaction, the resulting mixture was allowed to settle for phase separation. The bottom layer was removed which contained impurity, H_2_SO_4_, and excess methanol, while the top layer (esterified oil) was subjected to a transesterification reaction process after washing to remove the leftover acid.

**Table 4:** Acid value reduction for esterification of biodiesel.

Reaction temperature (°C)	Reaction time(min)	H_2_SO_4_ conc.(% v/v)	Acid value(mg KOH/ g oil)
–	–	–	4.10
50	40	1.0	3.70
60	50	1.5	3.40
70	60	2.0	2.80
80	70	2.5	2.50
90	80	3.0	2.02

### Transesterification: heterogeneous base treatment (calcined waste wood ash)

Synthesis of biodiesel from the esterified oil with the lowest acid value was carried out as follows: the reaction was carried out in a three-necked batch reactor flask. 120 ml of esterified oil was preheated at 70 °C for 60 min, 2.0 (wt.%) calcined waste wood ash (CWWA) was added to the flask containing 50 ml methanol (MeOH), the mixture was partially soluble and was homogenized by using a magnetic shaker The partially soluble preheated esterified oil mixture was adjusted to a reaction temperature of 60 °C for completion of the reaction. At the end of the reaction, the products were left to stand for 24 h to cool and to enhance phase separation. The used CWWA was recovered from biodiesel by decantation, while the methanol phase was separated from biodiesel by separating funnel through gravity settling. The leached CWWA in the biodiesel was removed through washing with the hot mixture of 2.0 g Na_2_CO_3_ dissolved in 40 ml methanol. The washed mixture was filtered and then washed with distilled water twice before separation over gravity settling. The wet biodiesel was further dried over anhydrous CaCl_2_ and filtered to obtain dry biodiesel. The percentage of biodiesel yield was calculated using Eq. (4). These steps were repeated according to the number of experimental runs (16) generated by Hybrid design (HD).

(4) $$BY\;\left(\%\;wt.\right)=\frac{Weight\;of\;pure\;biodiesel\;obtained}{\;weight\;of\;blended\;oil\;used}\;X\;100$$

Here, BY represents the experimental biodiesel yield.

### Experimental design for transesterification of biodiesel

For transesterification of esterified blended oil to biodiesel, four variable factors were considered for process modeling and optimization, this includes reaction time, reaction temperature, CWWA weight (wt.%), and methanol/oil molar ratio (CH_3_OH/OMR), respectively. A Hybrid Design (HD) with a minimal point design for 3, 4, 6, and 7 factors with 5 levels each were used for experimental design, which produced a total of sixteen experimental runs (16 runs) without repetitions. Table 5 displayed the five-level-four-variable- factors, the units, and the symbol used for the HD design (Table 6).

**Table 5:** Experimental design variables for transesterification.

Variables	Units	Symbol	Levels				
			−2	−1	0	1	2
Reaction time	(min)	$X_{1}$	40	45	50	55	60
Reaction temp.	(°C)	$X_{2}$	50	60	70	80	90
CWWA	(wt.%)	$X_{3}$	1.0	1.5	2.0	2.5	3.0
CH_3_OH/OMR	(vol./vol.)	$X_{4}$	3	4	5	6	7

**Table 6:** Biodiesel yield, predicted and residual values of transesterification of esterified oil.

Std	Run	X_1_ (min)	X_2_ (°C)	X_3_(% wt.)	X_4_ (vol./vol.)	BY (%v/v)	PBY (%v/v)	σ
1	9	0.000	0.000	0.000	1.732	92.00	92.00	0.000
2	4	0.000	0.000	0.000	−0.269	91.00	91.00	0.000
3	16	−1.000	−1.000	−1.000	0.604	93.43	93.42	2.500E-003
4	3	1.000	−1.000	−1.000	0.604	93.57	93.58	−2.500E-003
5	15	−1.000	1.000	−1.000	0.604	95.17	95.18	−2.500E-003
6	10	1.000	1.000	−1.000	0.604	96.62	96.62	2.500E-003
7	11	−1.000	−1.000	1.000	0.604	92.68	92.68	−2.500E-003
8	2	1.000	−1.000	1.000	0.604	94.42	94.42	2.500E-003
9	13	−1.000	1.000	1.000	0.604	95.82	95.82	2.500E-003
10	1	1.000	1.000	1.000	0.604	98.82	98.82	−2.500E-003
11	5	1.518	0.000	0.000	−1.050	98.76	98.75	0.001
12	14	−1.518	0.000	0.000	−1.050	96.00	96.00	0.000
13	7	0.000	1.518	0.000	−1.050	99.14	99.15	−0.001
14	6	0.000	−1.518	0.000	−1.050	91.22	91.20	0.000
15	12	0.000	0.000	1.518	−1.050	96.59	96.60	−0.001
16	8	0.000	0.000	−1.518	−1.050	88.64	88.64	0.000

σ = residual value, X_1_ = reaction time, X_2_ = reaction temperature, X_3_ = catalyst weight, X_4_ = methanol/oil molar ratio, BY = biodiesel yield, PBY = predicted biodiesel yield.

### Statistical analysis of biodiesel production

**Fig. 3:**
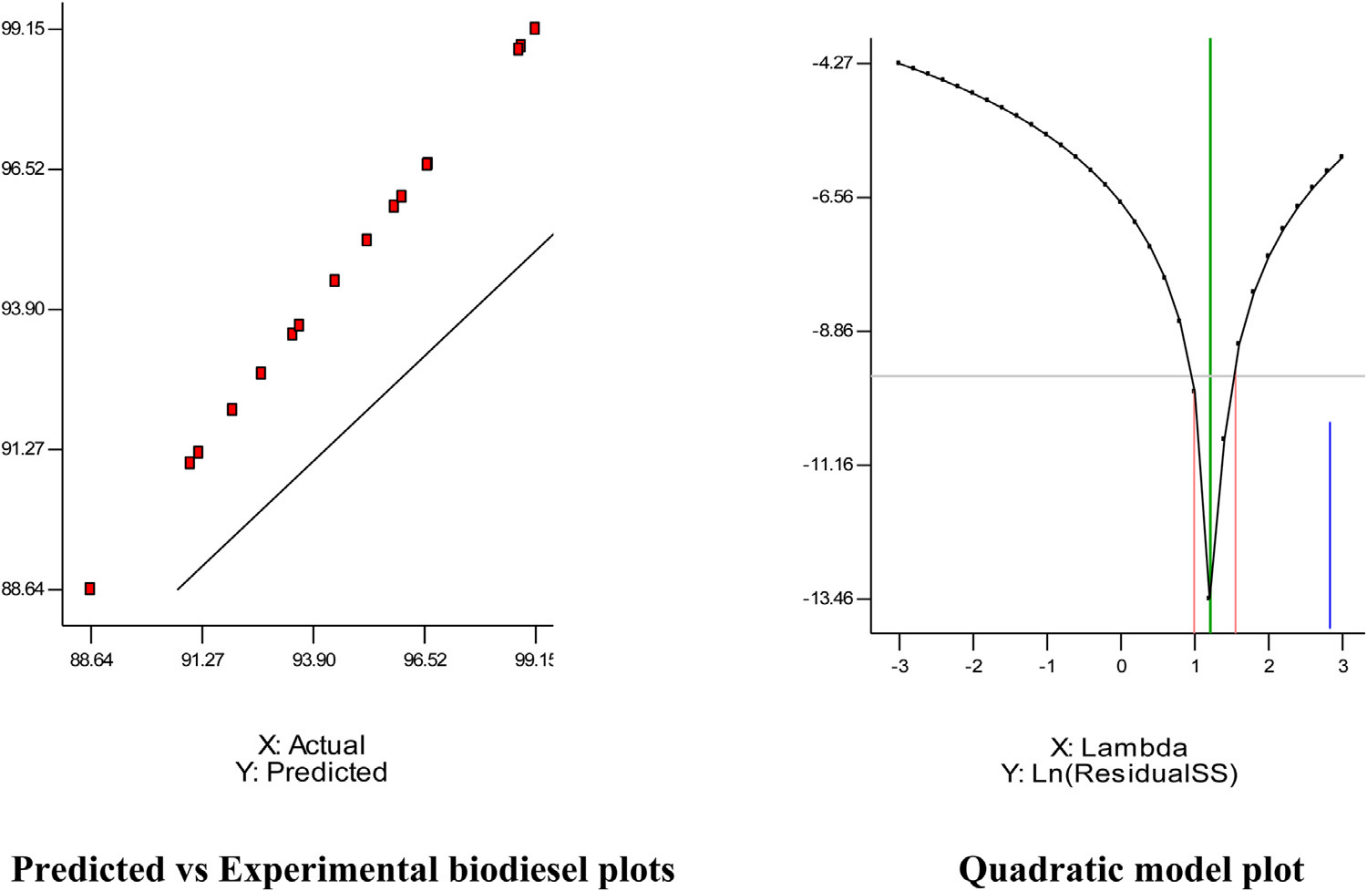
Model plots.

**Fig. 4:**
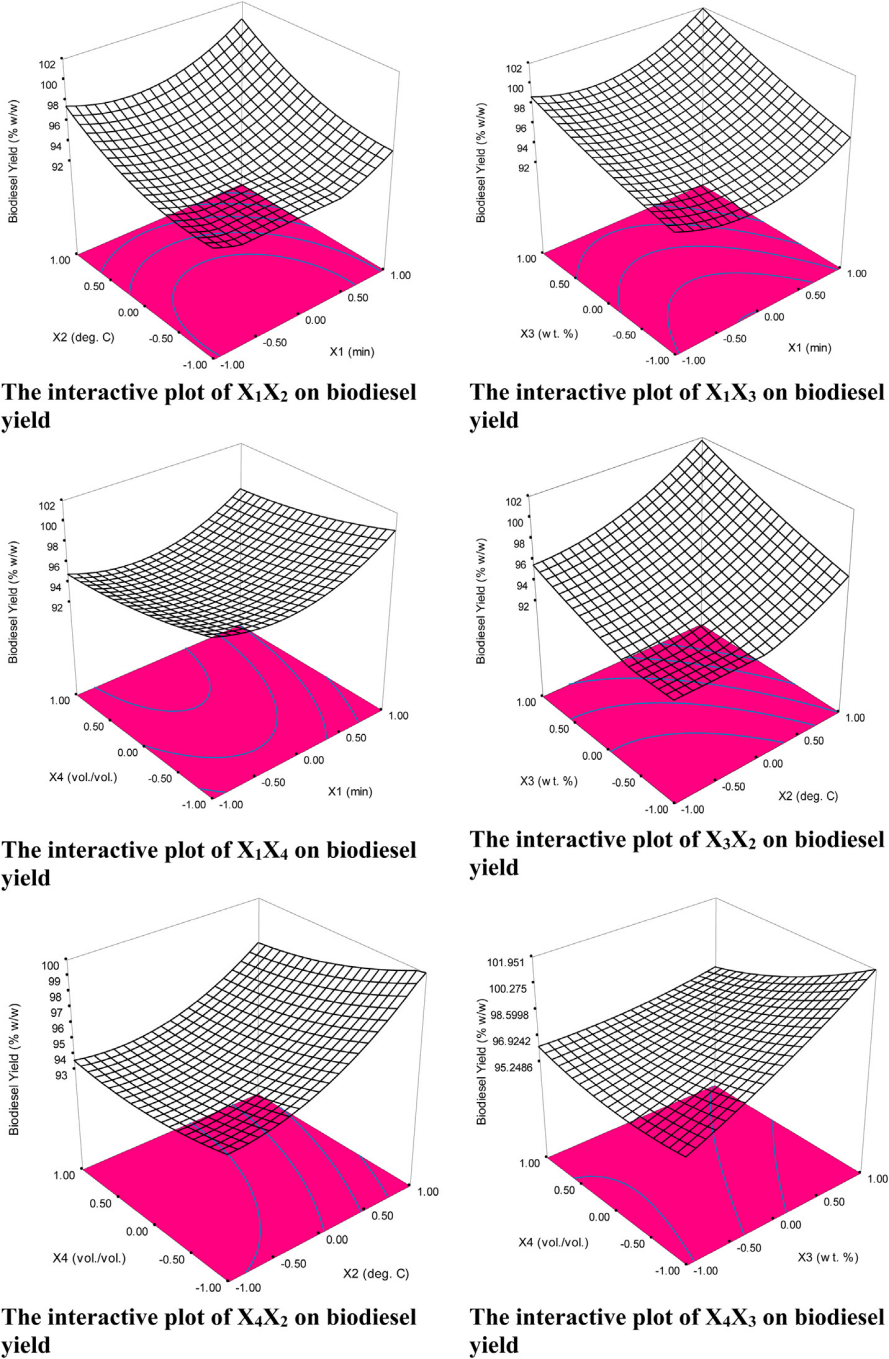
3-dimensional plots.

Statistical analysis of the transesterification process of biodiesel production was carried out based on experimental results and predicted values by the HD with the variables factors as the constraints via fit summary. The model second order was used to establish the relationship between the biodiesel yield and the four-variable factors. Analysis of Variance (ANOVA) was used to test the model significance and estimate the fit of the model depicted in Table 7. The contour and the three-dimensional plots were used to determine the interactive effects of the linear variables on the biodiesel yield as illustrated in Figs. 3 and 4. The probability value (p-value) and factor value (f-value), the variance inflation factor (VIF), and the degree of freedom (df) were also elucidated for model significance. The R-square coefficient of determination ($R^{2}),$ the R-square predicted coefficient of determination$\;(R_{pred.}^{2}$), and the R-square adjusted coefficient of determination ($R_{adj.}^{2}),\;$were estimated arithmetically and used to check the model suitability. The final model correlation relationship that expressed vividly the response for every change of variables concerning optimum yield was presented in terms of the second-order differential equation expressed as in Eq. (5).

**Table 7:** Anova and test of significant for biodiesel statistical analysis.

Source	Sum of Squares	df	Mean Square	F Value	Prob > F
Model	140.63	14	10.04	2.009E+005	0.0017
$X_{1}$	8.79	1	8.79	1.759E+005	0.0015
$X_{2}$	47.25	1	47.25	9.449E+005	0.0007
$X_{3}$	17.90	1	17.90	3.579E+005	0.0011
$X_{4}$	1.37	1	1.37	27,438.83	0.0038
$X_{1}^{2}$	35.29	1	35.29	7.059E+005	0.0008
$X_{2}^{2}$	12.79	1	12.79	2.557E+005	0.0013
$X_{3}^{2}$	0.70	1	0.70	13,943.38	0.0054
$X_{4}^{2}$	1.64	1	1.64	32,763.02	0.0035
$X_{1}X_{2}$	0.81	1	0.81	16,129.00	0.0050
$X_{1}X_{3}$	1.23	1	1.23	24,649.00	0.0041
$X_{1}X_{4}$	0.040	1	0.040	797.23	0.0225
$X_{2}X_{3}$	0.94	1	0.94	18,769.00	0.0046
$X_{2}X_{4}$	3.39	1	3.39	67,767.35	0.0024
$X_{3}X_{4}$	14.86	1	14.86	2.973E+005	0.0012
Residual	5.000E-005	1	5.000E-005		
Cor Total	140.63	15			

**Fits statistics**			
Std. Dev.	7.071E-003	R-Squared	0.9999
Mean	94.62	Adj R-Squared	0.9997
C.V.	7.473E-003	Pred R-Squared	0.9998
PRESS	N/A	Adeq Precision	1535.084

## The final equation in terms of coded factors

(5) $$\begin{array}{ccc} \text{BY}(\%\text{wt}.) & = & +90.87+0.84X_{1}+1.94X_{2}+1.19X_{3}-0.33X_{4}+0.32X_{1}X_{2}+0.39X_{1}X_{3}\\ & & -\;0.071X_{1}X_{4}+0.34X_{2}X_{3}-0.65X_{2}X_{4}-1.36X_{3}X_{4}+2.40X_{1}^{2}\;+\;1.45X_{2}^{2}\\ & & +\;0.34X_{3}^{2}\;+0.57X_{4}^{2} \end{array}$$

All the experiments were duplicated and the optimum predicted yield was validated in triplicate. The optimum yield (Table 8) was validated in triplicate, and average biodiesel yield was obtained.

**Table 8:** Optimum selected condition for biodiesel yield.

Number	X1 (min)	X2(deg. C)	X3(wt. %)	X4 (vol/vol)	Biodiesel Yield (% wt)	Desirability	
1	0.89	0.92	0.39	−0.78	99.1498	1.000	Selected
2	0.99	0.98	0.35	−0.46	99.1499	1.000	
3	0.95	0.93	0.38	−0.66	99.1502	1.000	
4	1.00	1.00	0.79	0.24	99.071	0.992	
5	−1.00	0.94	0.46	−1.00	97.898	0.881	
6	−1.00	1.00	0.34	−0.94	97.7156	0.864	
7	−0.99	1.00	1.00	−0.07	97.1149	0.806	
8	−1.00	1.00	−0.10	−1.00	96.7254	0.764	
9	−1.00	0.27	1.00	−1.00	96.5662	0.754	
10	0.99	−1.00	−1.00	1.00	94.5515	0.562	

### Quality of the biodiesel

The quality of the product was examined by determining the properties of the biodiesel such as cetane number, higher heating value, density, viscosity, moisture content, peroxide, saponification, and iodine value were determined via [22], and the values were compared with biodiesel recommended standards [23,24] (Table 9).

**Table 9:** Quality of mixed oil and biodiesel.

Parameter	Mixed oil	Biodiesel	astm d6751	en 14,214
Color@ 27 oc	yellowish-brown	brownish	–	–
Density (kg/m^3^) @ 25 °C	880	860	–	860–900
Viscosity @ 40 °C/ (mm^2^/s)	3.35	2.02	1.9–6.0	3.5–5.0
Moisture content (%)	0.001	<0.001	<0.03	0.02
%FFA (as oleic acid)	1.01	0.21	0.40 max	0.25 max
Acid value (mg KOH/g oil)	2.02	0.42	0.80 max	0.50 max
Iodine value (g I_2_/100 g oil)	98.70	77.24	–	120 max
Saponification value (mg KOH/g oil)	214.30	120.20	–	–
Peroxide value (meq O_2_/kg oil)	1.40	1.20	–	12.85
HHV (MJ/kg)	39.16	43.34	–	–
Cetane number	49.56	74.33	57 min	51 min
API	29.30	33.03	39.95 max	–
Diesel index	54.94	89.35	50.4 min	–

### Performance estimation of CWWA

**Fig. 5:**
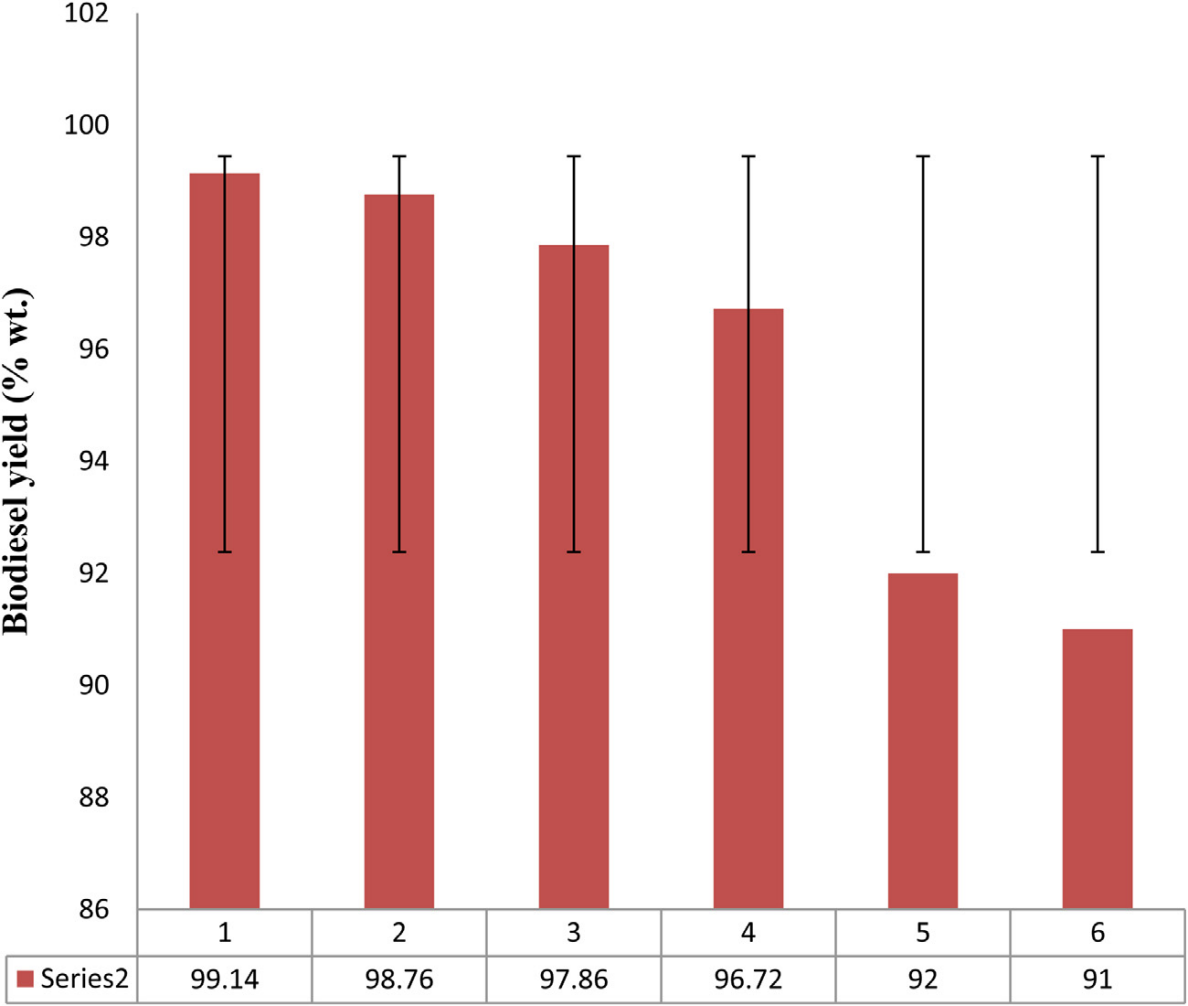
Reusability test plot.

The strength of the catalyst developed was tested through catalyst recyclability and reusability test. The CWWA was recycled at the end reaction, because of the impurity at the surface of the regenerated catalyst; the washing with alcohol to remove impurity was carried out before being centrifuged at 4000 rpm for 25 min, and then filtered to obtain wet CWWA. The wet CWWA was then oven-dried at 150 °C for 120 min, allowed to cool at room temperature before reusing. The degeneration in catalyst strength was established by graphical plot, with a reduction in 5th and 6th cycles. Henceforward, the catalyst reusability test was stopped at the 4th cycle (Fig. 5).

### Analysis of spent catalyst

Analysis of the spent catalyst was studied after reusability and recyclability to depict its morphological characteristic by BET and the effect of reaction on the CaO by XRD. Observation showed that the S_BET_ (Surface area) and the total pore volume increased as the reusability continued, while the basic site (BS), the total basic site (TBS), and the basic site density (BSD) of the spent catalyst decreased (Table 10). This is an indication that catalyst recyclability, purification, and reusability decreased the basic strength of the catalyst during the reaction process. The main aim of catalyst regeneration is targeted at restoring the high surface area and high dispersal of the element in the catalysts [26]. Furthermore, the effect of leaching within the reactor can easily lead to coverage of active surface sites hereby decreasing the biodiesel yield. The results displayed by XRD analysis alongside with the BET displayed that the percentage decrease to (25.43%) in CaO derived from CWWA was very high after reusability and regeneration. It was observed that the percentage of CaO decreased from 62.83% to 25.43%.

**Table 10:** Characteristic of a spent CWWA.

Catalyst	N_2_-AA(m^2^g^−1^)	TPV(cm^3^g^−1^)	%CaO	BS (μmole.g^−1^)400<BS<500 >500		TBS	BSD (μmole/m^2^)
*CWWA*	1.40	0.0080	25. 43	–	86.00	86.00	61.43

N_2_-AA= nitrogen adsorption analysis, TPV = Total pore volume, BS = Basic site, TBS = Total basic site, BSD = Basic site density.

## Appendix Supplementary materials

Supplementary Materials: Table and Figure**[COMP: Plz. check suppl. material]** Image, application 1
