# Supplementary material for: Synthesis of biodiesel from Annona muricata – Calophyllum inophyllum oil blends using calcined waste wood ash as a heterogeneous base catalyst
Source: MethodsX. 2020 Dec 23;8:101188. doi: 10.1016/j.mex.2020.101188 (PMC8374236; doi:10.1016/j.mex.2020.101188)
Supplement: Supplementary file 1 — Supplementary Materials: Table and Figure[COMP: Plz. check suppl. material] [file mmc1.docx]

**Properties of oils and Blended oil**

| **Properties** | **AMO** | **CIO** | **Mixed/Blended oil** | **Total API gravity** |
| --- | --- | --- | --- | --- |
| Moisture content (%) | 0.011 | 0.011 | 0.001^a^ |  |
| Viscosity @ 40 ^o^C/ (mm^2^/s) | 1.32 | 2.40 | 1.86^a^ |  |
| Acid value (mg KOH/g oil) | 1.56 | 6.84 | 4.10^a^ |  |
| % Free Fatty Acid (FFA) | 0.78 | 2.92 | 2.05^a^ |  |
| Peroxide value (meq O_2_/kg oil) | 1.34 | 1.40 | ND |  |
| Saponification value (mg KOH/g oil) | 224.63 | 201.00 | ND |  |
| Iodine value (g I_2_/100g oil) | 114.32 | 68.56 | ND |  |
| Specific gravity | 0.82 | 0.91 | ND |  |
| API gravity | 41.05 | 24.00 | ND |  |
| API gravity ratio (%) | 63 | 37 |  |  |
| Simplest Ratio | 1.7 | 1 |  |  |
| **Blended ratio** | **AMO_63_ : CIO_37_** | |  |  |

where, am = Value after mixed/blended, NYD = Not Determined


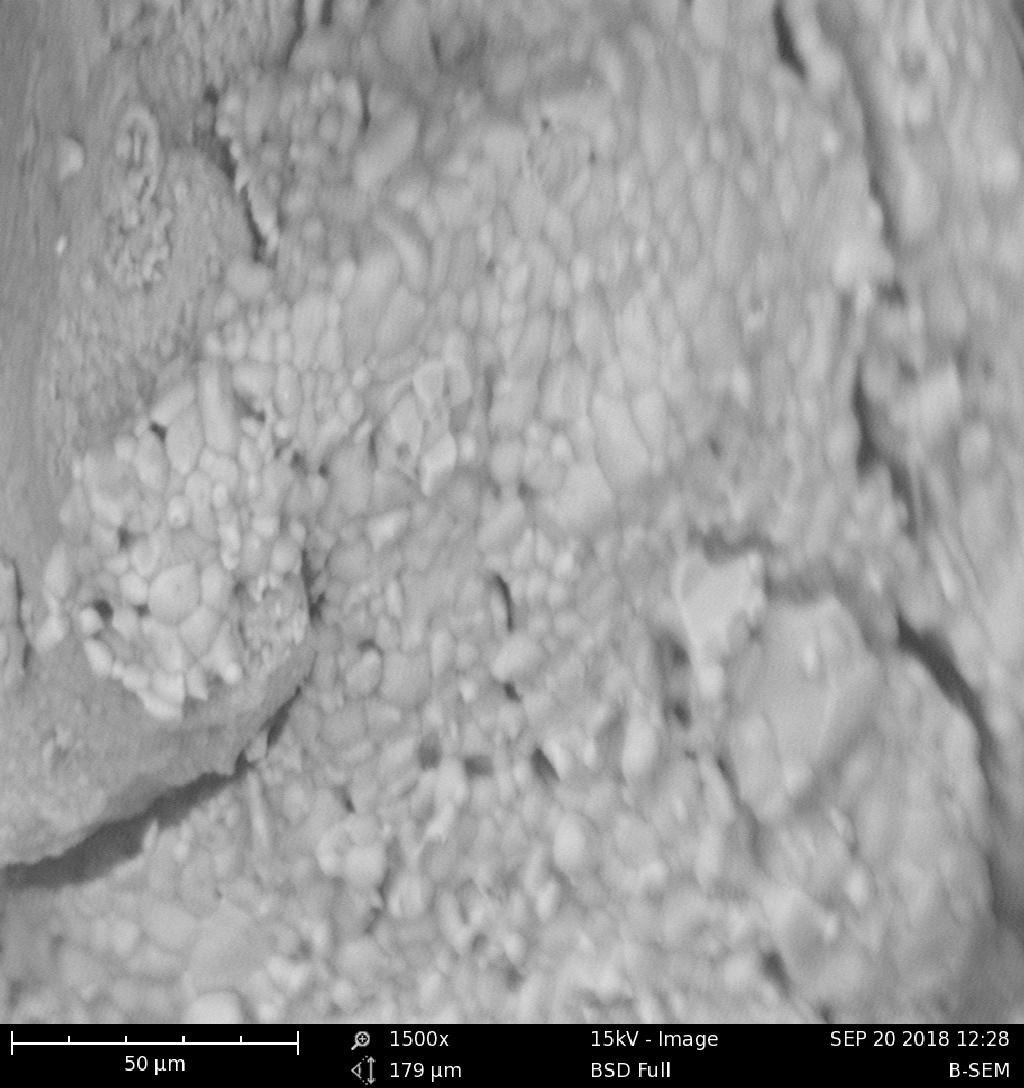

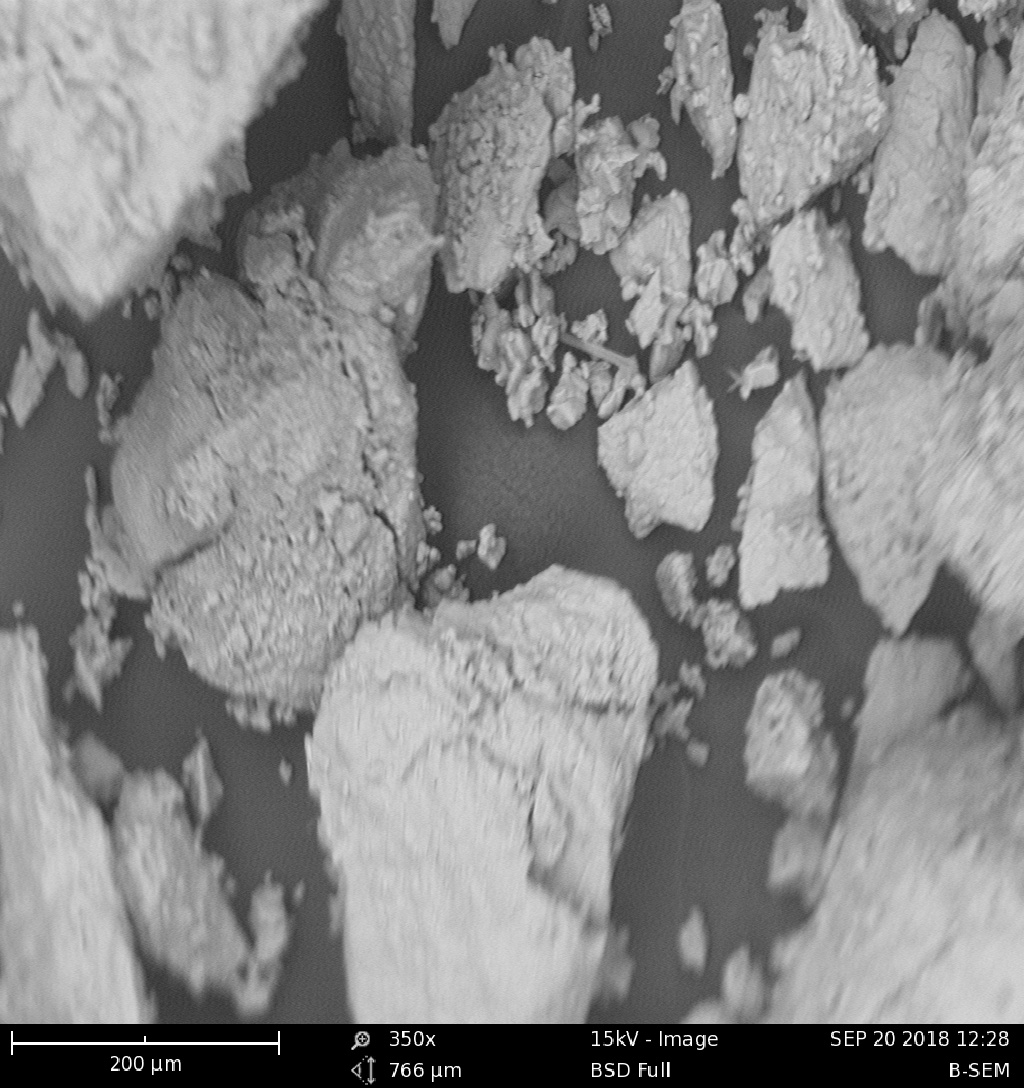


**SEM After Calcination**

**SEM Before Calcination**

**SEM Analysis of the WWA**

**FTIR spectral of CWWA**

**FTIR sample spectrum analysis of CWWA**

| **SN** | **Wavelength (cm^-1^ )** | **Transmittance (%)** | **Bonding Functional groups** |
| --- | --- | --- | --- |
|  | | | |
| 1 | 760.4 to 1077.2 | 71.799 to 69.142 | C-Cl, CO_3_^2-^, N-H waging and twisting, O=C=O bending vibration |
| 2 | 1148.0 to 1636.3 | 69.142 to 81.972 | C-C, C=C, C=N, C=O, CHO, C$\equiv$C, C$\equiv$N, O=C=O of low energy, O-H, and O-Ca-O bending vibration |
| 3 | 2928.0 to 3272.0 | 83.297 to 74.128 | O-H bending structure, O=O, and N$\equiv$O |

**BET-adsorption, XRD analysis and Hammett indicator value of calcined CWWA**

| **Catalyst** | **N_2_-AA**  **(m^2^g^-1^)** | **TPV**  **(cm^3^g^-1^)** | **%CaO** | **BS (μmole.g^-1^)**  400<BS<500 >500 | | **TBS** | **BSD (μmole/m^2^)** |
| --- | --- | --- | --- | --- | --- | --- | --- |
| *CWWA* | 1.10 | 0.0045 | 62.83 | 47 | 162 | 209 | 190.00 |

N_2_-AA= nitrogen adsorption analysis, TPV = Total pore volume, BS = Basic site, TBS = Total basic site, BSD = Basic site density

**Acid value reduction for esterification of biodiesel**

| **Reaction temperature (^o^C)** | **Reaction time**  **(min)** | **H_2_SO_4_ conc.**  **(% v/v)** | **Acid value**  **(mg KOH/ g oil)** |
| --- | --- | --- | --- |
| - | - | - | 4.10 |
| 50 | 40 | 1.0 | 3.70 |
| 60 | 50 | 1.5 | 3.40 |
| 70 | 60 | 2.0 | 2.80 |
| 80 | 70 | 2.5 | 2.50 |
| 90 | 80 | 3.0 | 2.02 |

**Biodiesel yield, predicted and residual values of transesterification of esterified oil**

| **Std** | **Run** | **X_1_ (min)** | **X_2_ (^o^C)** | **X_3_**  **(% wt.)** | **X_4_ (vol./vol.)** | **BY (%v/v)** | **PBY (%v/v)** | $\boldsymbol{\sigma}$ |
| --- | --- | --- | --- | --- | --- | --- | --- | --- |
| 1 | 9 | 0.000 | 0.000 | 0.000 | 1.732 | 92.00 | 92.00 | 0.000 |
| 2 | 4 | 0.000 | 0.000 | 0.000 | -0.269 | 91.00 | 91.00 | 0.000 |
| 3 | 16 | -1.000 | -1.000 | -1.000 | 0.604 | 93.42 | 93.42 | 2.500E-003 |
| 4 | 3 | 1.000 | -1.000 | -1.000 | 0.604 | 93.58 | 93.58 | -2.500E-003 |
| 5 | 15 | -1.000 | 1.000 | -1.000 | 0.604 | 95.18 | 95.18 | -2.500E-003 |
| 6 | 10 | 1.000 | 1.000 | -1.000 | 0.604 | 96.62 | 96.62 | 2.500E-003 |
| 7 | 11 | -1.000 | -1.000 | 1.000 | 0.604 | 92.68 | 92.68 | -2.500E-003 |
| 8 | 2 | 1.000 | -1.000 | 1.000 | 0.604 | 94.42 | 94.42 | 2.500E-003 |
| 9 | 13 | -1.000 | 1.000 | 1.000 | 0.604 | 95.82 | 95.82 | 2.500E-003 |
| 10 | 1 | 1.000 | 1.000 | 1.000 | 0.604 | 98.82 | 98.82 | -2.500E-003 |
| 11 | 5 | 1.518 | 0.000 | 0.000 | -1.050 | 98.76 | 98.76 | 0.000 |
| 12 | 14 | -1.518 | 0.000 | 0.000 | -1.050 | 96.00 | 96.00 | 0.000 |
| 13 | 7 | 0.000 | 1.518 | 0.000 | -1.050 | 99.15 | 99.15 | 0.000 |
| 14 | 6 | 0.000 | -1.518 | 0.000 | -1.050 | 91.20 | 91.20 | 0.000 |
| 15 | 12 | 0.000 | 0.000 | 1.518 | -1.050 | 96.60 | 96.60 | 0.000 |
| 16 | 8 | 0.000 | 0.000 | -1.518 | -1.050 | 88.64 | 88.64 | 0.000 |

$\sigma$ = residual value, X_1_ = reaction time, X_2_ = reaction temperature, X_3_ = catalyst weight, X_4_ = methanol/oil molar ratio, BY = biodiesel yield, PBY = predicted biodiesel yield

**Anova and test of significant for biodiesel statistical analysis**

| **Source** | **Sum of Squares** | **dF** | | **Mean Square** | **F Value** | | **Prob > F** |
| --- | --- | --- | --- | --- | --- | --- | --- |
| Model | 140.63 | 14 | | 10.04 | 2.009E+005 | | 0.0017 |
| $X_{1}$ | 8.79 | 1 | | 8.79 | 1.759E+005 | | 0.0015 |
| $X_{2}$ | 47.25 | 1 | | 47.25 | 9.449E+005 | | 0.0007 |
| $X_{3}$ | 17.90 | 1 | | 17.90 | 3.579E+005 | | 0.0011 |
| $X_{4}$ | 1.37 | 1 | | 1.37 | 27438.83 | | 0.0038 |
| $X_{1}^{2}$ | 35.29 | 1 | | 35.29 | 7.059E+005 | | 0.0008 |
| $X_{2}^{2}$ | 12.79 | 1 | | 12.79 | 2.557E+005 | | 0.0013 |
| $X_{3}^{2}$ | 0.70 | 1 | | 0.70 | 13943.38 | | 0.0054 |
| $X_{4}^{2}$ | 1.64 | 1 | | 1.64 | 32763.02 | | 0.0035 |
| $X_{1}X_{2}$ | 0.81 | 1 | | 0.81 | 16129.00 | | 0.0050 |
| $X_{1}X_{3}$ | 1.23 | 1 | | 1.23 | 24649.00 | | 0.0041 |
| $X_{1}X_{4}$ | 0.040 | 1 | | 0.040 | 797.23 | | 0.0225 |
| $X_{2}X_{3}$ | 0.94 | 1 | | 0.94 | 18769.00 | | 0.0046 |
| $X_{2}X_{4}$ | 3.39 | 1 | | 3.39 | 67767.35 | | 0.0024 |
| $X_{3}X_{4}$ | 14.86 | 1 | | 14.86 | 2.973E+005 | | 0.0012 |
| Residual | 5.000E-005 | 1 | | 5.000E-005 |  | |  |
| Cor Total | 140.63 | 15 | |  |  | |  |
| **Fits statistics** | | | | | | | |
| Std. Dev. | 7.071E-003 | | R-Squared | | | 0.9999 | |
| Mean | 94.62 | | Adj R-Squared | | | 0.9997 | |
| C.V. | 7.473E-003 | | Pred R-Squared | | | 0.9998 | |
| PRESS | N/A | | Adeq Precision | | | 1535.084 | |

**Final Equation in Terms of Coded Factors:**

$$\mathrm{BY}\left( \%wt. \right)=+90.87+0.84X_{1}+1.94X_{2}+1.19X_{3}-0.33X_{4}+0.32X_{1}X_{2}+0.39X_{1}X_{3}$$

${-0.071X}_{1}X_{4}+0.34X_{2}X_{3}-{0.65X}_{2}X_{4}-1.36X_{3}X_{4}+2.40X_{1}^{2}+1.45X_{2}^{2}+0.34X_{3}^{2}+0.57X_{4}^{2}$

**Predicted vs Experimental biodiesel plots Quadratic model plot**

|   **Interactive plot of X_1_X_2_ on biodiesel yield** |   **Interactive plot of X_1_X_3_ on biodiesel yield** |
| --- | --- |
|  **Interactive plot of X_1_X_4_ on biodiesel yield** |   **Interactive plot of X_3_X_2_ on biodiesel yield** |
|   **Interactive plot of X_4_X_2_ on biodiesel yield** |  **Interactive plot of X_4_X_3_ on biodiesel yield** |
| **3-Dimensional plots** | |

**Optimum selected condition for biodiesel yield**

| **Number** | **X1 (min)** | **X2**  **(deg. C)** | **X3**  **(wt. %)** | **X4 (vol/vol)** | **Biodiesel Yield (% wt)** | **Desirability** |  |
| --- | --- | --- | --- | --- | --- | --- | --- |
| 1 | 0.89 | 0.92 | 0.39 | -0.78 | 99.1498 | 1.000 | **Selected** |
| 2 | 0.99 | 0.98 | 0.35 | -0.46 | 99.1499 | 1.000 |  |
| 3 | 0.95 | 0.93 | 0.38 | -0.66 | 99.1502 | 1.000 |  |
| 4 | 1.00 | 1.00 | 0.79 | 0.24 | 99.071 | 0.992 |  |
| 5 | -1.00 | 0.94 | 0.46 | -1.00 | 97.898 | 0.881 |  |
| 6 | -1.00 | 1.00 | 0.34 | -0.94 | 97.7156 | 0.864 |  |
| 7 | -0.99 | 1.00 | 1.00 | -0.07 | 97.1149 | 0.806 |  |
| 8 | -1.00 | 1.00 | -0.10 | -1.00 | 96.7254 | 0.764 |  |
| 9 | -1.00 | 0.27 | 1.00 | -1.00 | 96.5662 | 0.754 |  |
| 10 | 0.99 | -1.00 | -1.00 | 1.00 | 94.5515 | 0.562 |  |
